# Dietary Inclusion of *Halobacterium salinarum* Modulates Growth Performances and Immune Responses in Farmed Gilthead Seabream (*Sparus aurata* L.)

**DOI:** 10.3390/ani13172743

**Published:** 2023-08-29

**Authors:** Concetta Maria Messina, Manfredi Madia, Simona Manuguerra, Cristobal Espinosa-Ruiz, María Angeles Esteban, Andrea Santulli

**Affiliations:** 1 Laboratory of Marine Biochemistry and Ecotoxicology, Department of Earth and Marine Sciences DiSTeM, University of Palermo, Via Barlotta 4, 91100 Trapani, Italy; madiamanfredibio@gmail.com (M.M.); simona.manuguerra@unipa.it (S.M.); andrea.santulli@unipa.it (A.S.); 2 Department of Cell Biology and Histology, Faculty of Biology, Campus Regional de Excelencia Internacional Campus Mare Nostrum, University of Murcia, 30100 Murcia, Spain; cer48658@um.es (C.E.-R.); aesteban@um.es (M.A.E.); 3 Istituto di Biologia Marina, Consorzio Universitario della Provincia di Trapani, Via G. Barlotta 4, 91100 Trapani, Italy

**Keywords:** aquaculture-related species, archaea, humoral and cellular immune parameters, bactericidal activity

## Abstract

**Simple Summary:**

It is known that, in intensive farming, fish are often exposed to multiple sources of stress that increase their susceptibility to pathogens and promote disease outbreaks. The latter are recognized as a major constraint on sustainable animal production, which can cause significant economic losses in the aquaculture sector. Many drugs have traditionally been used for the treatment and prevention of diseases in farmed fish, but they are not recommended, because the continued overuse of antibiotics can lead to the development of antibiotic-resistant bacteria, environmental pollution and the accumulation of toxic residues in fish. Over the past decade, scientific research has focused on the isolation, identification and use of bioactive compounds (rich in carotenoids and other natural antioxidants) obtained from microalgae, plants and micro-organisms, with the goal to develop alternative dietary supplements that improve the growth performance, health and immune system of farmed fish. The reinforcement of fish defense mechanisms through the prophylactic administration of immunostimulants, probiotics, prebiotics and other natural substances is one of the most promising methods to improve the profitability of aquaculture by improving the immune response of fish and decreasing the risk associated with the use of chemicals.

**Abstract:**

The use of natural immunostimulants is considered the most promising alternative to promote fish health, productive performance and quality, increasing the aquaculture profitability, sustainability and social acceptance. The purpose of this study was to evaluate the effect of the integration of a potential probiotic strain, *Halobacterium salinarum*, belonging to the Archaea domain, in the formulated diets of farmed gilthead seabream (*Sparus aurata* L.) in terms of growth performances and immunity responses. The experiment was set up to test two different levels of inclusion of the bacteria in the diet: 0.05% (D1) and 0.1% (D2). The effects on fish growth performances; humoral (peroxidase, protease, antiprotease and IgM levels) and cellular immunity parameters (phagocytosis, respiratory burst and myeloperoxidase), along with bactericidal activity, were evaluated after 15 and 30 days of experimental feeding. The obtained results showed that the inclusion of *H. salinarum* at the highest concentration (D2 0.1%) improved growth performances, bactericidal activity against *Vibrio anguillarum* and some parameters related both to the humoral and cellular immune response, suggesting exploring other aspects of welfare in view of future supplementations of this probiotic strain in the diet of *S. aurata*.

## 1. Introduction

Aquaculture expansion is currently facing challenges depending on farmed species diversification, the improvement and optimization of the diets of reared animals and diseases control [1,2]. One of the most important areas of aquaculture sector is the production of sustainable, cost-effective commercial diets able to promote fish growth and health, contributing to the production of high-quality product for consumers [3,4].

The attention to fish welfare issues is determined by the multiple sources of stress in intensive farming that could compromise their immune systems, increasing fish susceptibility to pathogens and promoting disease outbreaks, with negative effects on quality and production performances. Confinement, high stocking density, chemical parameters of water, handling and transport are common procedures that can induce stress in farmed fish, and a chronic or long-term exposure to these stressors generally has suppressive effects on the immune system and disease resistance of fish [5,6].

Traditionally, disease control and prevention strategies in aquaculture have relied on the use of vaccines, antibiotics and chemotherapeutics [7]; however, the administration of antibiotics can promote the development of resistant bacterial strains and even adversely affect the health status of fish, as well as their degradation can have an environmental impact [8,9]. For this reason, the use antibiotics as feed additive are highly controlled and monitored in the European Union (European Parliament and Council Regulation EC No. 1831/2003), limiting its utilization only to necessary treatments in order to ensuring safety of aquaculture products [10]. In view of these directives, there has been an increasing interest in Europe to promote the inclusion of natural compounds in aquafeeds in order to strengthen the immune status of fish, decreasing the risk associated with the use of chemicals [5,11]. 

The prophylactic administration of immunostimulants, probiotics, prebiotics and other natural substances is one of the most promising methods to improve the health and growth performance of fish and, consequently, the aquaculture profitability [9,12]. 

However, feed processing technologies, e.g., temperature and pressure, have a negative impact on the viability of conventional probiotics due to the reduction of their potential bioactivity and, consequently, effectiveness in aquaculture [10]. As routine industrial heat treatments are capable of destroying microbes and microbial-based products, reducing their potential and functionality [13], researchers are focusing on the compatibility of feed probiotics with industrial processing involving the utilization of high temperatures and pressures [14]. In this regard, recently, some studies have therefore focused on the possible use of unconventional organisms, such as Archaea, as feed additives in fish diets to improve the immunity of fish [15,16]. Archaea belong to a third domain and present unusual characteristics, such as a tolerance to pressure, salt and temperature variability [10,17]; moreover, as one of the gut microbial communities that participate in various animal health-oriented biological functions, they could represent a potential for the aquaculture industry [10,15]. These organisms have a high resistance to industrial processing compared to classical probiotic organisms, where industrial processing remains the biggest challenge [18]. It was reported that Archaea activate B and T cells in the host, inducing adaptive immune responses [10], and they are involved in reducing inflammation through systemic immune responses [10,15,16].

As Archaea belong to fish gut microbiota [10], they can be used as ‘archaebiotics’ to improve the performance of fish health. However, the mode of action in fish guts and the specific interactive mechanisms between Archaea and the host immune system need to be investigated. 

Halophilic archaea (haloarchaea or, traditionally, ‘halobacteria’), belonging to the family of *Halobacteriacaea*, exhibit a range of stress responses to counterbalance the deleterious effects induced by the changing of environmental parameters. In particular, *H. salinarum* accumulates a heat shock protein in the cytosol (the so called ‘thermosome;), known to stabilize proteins during thermal stress [17,19], produce and accumulate carotenoids, such as lycopene and beta carotene, which are responsible for their red color, and have a considerable potential for application as nutraceuticals and dietary antioxidants due to their antioxidant and pro-vitamin properties [20,21,22].

In view of this, the aim of the present study was to investigate, for the first time, the effects of inclusion of *Halobacterium salinarum* in the diet of gilthead seabream (*Sparus aurata*) at two different doses (D1 0.1% and D2 0.05%) in terms of the growth performance, bactericidal activity and immunity response at the humoral and cellular levels after 15 and 30 days of treatment.

## 2. Materials and Methods

### 2.1. Fish

Forty-two individuals of gilthead seabream (*S. aurata*) with a mean body weight of 8 ± 1.07 g were obtained from a local farm (Murcia, Spain) and distributed in 7 aquaria (250 L capacity) equipped with an aeration system, biological and active carbon filters and a closed water circuit with a flow rate of 900 L/h. The parameters and quality of the water were monitored weekly and kept constant. Thus, the pH was maintained between 7.5 and 8.0, 28‰ salinity, the water temperature between 20 and 22 °C and the photoperiod was 12 h light/12 h dark. The ammonia and nitrite concentrations were ≤0.1 mg L^−1^ and ≤0.2 mg L^−1^, respectively. During the experimental period, the animals were fed at a rate of 1% of the body weight per day^−1^. 

### 2.2. Preparation of Diets

For the preparation of the experimental diets, a commercial feed specific for gilthead seabream was selected as a base (D-2 Optibream AE 1P, Skretting), grinded and supplemented with lyophilized *H. salinarum* in order to obtain two levels of integration: 0.05% (treatment D1, with 5 × 10^8^ CFU/g diet) and 0.1% (treatment D2, with 1 × 10^9^ CFU/g diet), according to Díaz-Rosales et al. [23]. The mixture was stirred, water was added and mixing was continued with a mixer in order to obtain a compact mass from which feed grains of a similar size to the starting size were obtained. The feed was left to dry and stored at 4 °C for the duration of the experiment.

### 2.3. Experimental Design and Sampling 

The control diet and the experimental diets, supplemented with *H. salinarum* at 0.05% (D1) and 0.1% (D2), were administered for one month. For the experiment, 3 tanks were allowed for control and 2 tanks for each dose tested at each sampling time, each with 6 fish.

The animals were fed once a day with a ratio equal to one percent of the body weight. Fish were sampled at time zero (T0) at 15 and 30 days. For each time, two individuals were sampled from the three control tanks (n = 6), while, at T15 and T30, three individuals were sampled from each tank of the D1and D2 treatments (n = 6).

The experiment complied with the EU Directive 2010/63/EU for animal experiments. Animals were sacrificed by an overdose of MS222 (100 mg L^−1^; Sandoz, Holzkirchen, Germany), and samples of blood, skin mucus and head kidney (HK) were obtained from each individual. The skin mucus was collected from all fish using the routine methodology [5], gently scraping the surface of the fish using a plastic cell scraper while avoiding contamination with blood and urine–genital or intestinal excretions. The mucus was centrifuged (10,062× *g*, 4 °C, 10 min), and the supernatants were immediately stored at −80 °C until analysis. Blood samples were collected from the caudal vein with an insulin syringe of 1 mL and were left to clot at 4 °C for 4 h. After centrifugation (10,062× *g*, 10 min, 4 °C), the serum was collected and stored at −80 °C.

HK leucocytes were isolated to investigate the cellular immune activities [24]. Briefly, HK were taken, cut into small fragments, passed through a cell strainer (100 μm nylon) and transferred to 10 mL of sRPMI (RPMI-1640 culture medium (Sigma-Aldrich, St. Louis, MO, USA) supplemented with 0.35% sodium chloride, 2% fetal calf serum (Sigma-Aldrich, St. Louis, MO, USA), 10 μg mL^−1^ heparin (Sigma-Aldrich, St. Louis, MO, USA), 100 IU mL^−1^ penicillin (Flow, Sigma-Aldrich, St. Louis, MO, USA) and 100 mg mL^−1^ streptomycin (Flow, Sigma-Aldrich, St. Louis, MO, USA). Then, HK leucocytes were washed twice (400 g, 10 min) with sRPMI (without heparin) and counted (Z2 Coulter Particle Counter, Beckman Coulter, Barcelona, Spain) to adjust the viable cells at 10^7^ cells mL^−1^ using the trypan blue exclusion test.

### 2.4. Growth Performance 

The body weight of each fish was measured before and after the trial, and growth was monitored by obtaining the weight gain (WG%) and the specific growth rate (SGR, %day^−1^), calculated according to Silva–Carrillo et al. [25] (1):

WG% = [(final weight − initial weight)/initial weight] × 100 (1)

SGR%day^−1^ = [ln (final weight) − ln (initial weight) × 100]/days

### 2.5. Humoral Immune Parameters

#### 2.5.1. Peroxidase Activity 

The peroxidase activity in skin mucus was measured in all samples (n = 6 for each treatment at each time) according to Quade and Roth [26]. Briefly, 10 µL of skin and mucus were diluted with 40 µL of Hank’s buffer (HBSS) without Ca^+2^ or Mg^+2^, and for the serum, 5 µL were diluted with 45 µL of HBSS in flat-bottomed 96-well plates. As substrates, 100 µL of 10 mM tetramethylbenzidine (TMB; Sigma-Aldrich, St. Louis, MO, USA) and 5 mM H_2_O_2_ were added. The color change reaction was stopped after 2 min by adding 50 µL of 2 M H_2_SO_4_ (Sigma-Aldrich, St. Louis, MO, USA), and the OD was read at 450 nm on a plate reader (SpectostarNano, BMG Labtech, Ortenberg, Germany). Standard samples without skin mucus were used as blanks. One unit was defined as the amount producing an absorbance change of 1 and the activity expressed as U mL^−1^ of mucus.

#### 2.5.2. Protease Activity 

The protease activity was quantified in all samples (n = 6 for each treatment at each time), using the azocasein hydrolysis assay according to Ross et al. [27]. Briefly, 100 µL of skin mucus were incubated with 100 µL of 100 mM ammonium bicarbonate buffer containing 0.7% azocasein (Sigma-Aldrich, St. Louis, MO, USA) for 24 h at continuous shaking. The reaction was stopped by adding 4.6% trichloro acetic acid (TCA). Following centrifugation (19,722× *g* 5 min), the supernatants were transferred to a 96-well plate in duplicate, and 100 µL of 0.5 N NaOH were added to each well. The OD at 450 nm was measured using a plate reader. A positive control (100% of protease activity) and negative control (buffer, 0% activity) were used. For the serum protease, aliquots of 10 µL of serum were incubated with 100 µL of ammonium bicarbonate 100 mmol buffer containing 2% azocasein for 24 h of continuous shaking. The reaction was stopped by the addition of 10% TCA, and the experiment was continued as previously explained. 

#### 2.5.3. Antiprotease Activity

The total antiprotease activity was determined in all samples (n = 6 for each treatment at each time) by the ability to inhibit trypsin activity [26]. Aliquots of 10 µL of skin mucus were incubated (10 min, 22 °C) with the same volume of standard trypsin solution (5 mg mL^−1^). In the next step, 100 µL of 100 mM ammonium bicarbonate buffer and 125 µL of 2% azocasein were added, and the samples were incubated for 2 h with continuous shaking. Following this time, 250 µL of 10% TCA were added, and a final incubation (30 min) was achieved. The mixture was then centrifuged (19,722× *g* 5 min), with the supernatants transferred to a 96-well plate in duplicate containing 100 µL of 1 N NaOH and the OD read at 450 nm using a plate reader. For the positive control, a buffer replaced the skin mucus and trypsin, and for the negative control, a buffer replaced the skin mucus.

#### 2.5.4. Serum and Mucus IgM Levels

The total IgM levels in serum were analyzed in all samples (n = 6 for each treatment at each time) by ELISA (enzyme-linked immunosorbent assay) [28]. First, the serum obtained from the 36 samples was diluted 1:500, while, for the mucus, a dilution of 1:5 50 mM carbonate–bicarbonate buffer (pH 9.6) was used. Aliquots of 100 µL of the diluted serum or mucus were placed in triplicate in 96-well plates, and the proteins were fixed by incubating the plate overnight at 4 °C. This was followed by three 5 min washes with PBS-T (20 mM Tris-HCl, 150 mM NaCl and 0.05% Tween 20, pH 7.3), and the protein binding sites were blocked by incubation with a blocking buffer (PBS-T with 3% bovine serum albumin) for 2 h at room temperature. After washing again with PBS-T (three washes, 5 min, 200 µL), the plates were incubated for 1 h with 100 µL per well of the primary antibody, a mouse anti-gilthead seabream IgM (Aquatic Diagnostics Ltd., Stirling, Scotland) (1/100 in blocking buffer). After washing again, the plates were incubated with the secondary antibody (anti-mouse IgG-HRP, 1/1000 in blocking buffer). The plates were then washed thoroughly with PBS-T and developed with 100 µL of a 0.42 mM solution of TMB (Sigma) and 0.01% H_2_O_2_ prepared at the time of use. The reaction was incubated for 10 min and stopped by the addition of 50 µL of 2 M H_2_SO_4_. The plates were read at 450 nm. The negative controls consisted of samples without serum or without a primary antibody, which absorbance values was subtracted from the value of each sample.

### 2.6. Bactericidal Activity

Two fish pathogenic bacteria, (*Vibrio harveyi* and *Vibrio anguillarum*) were used in the bactericidal assay. All bacterial strains were grown from 1 mL of stock culture that had been previously frozen at −80 °C. The two bacteria were grown for 48 h at 25 °C in Tryptic Soy Agar (TSA, Difco Laboratories, Detroit, MI, USA) and then inoculated into Tryptic Soy Broth (TSB, Difco Laboratories, Detroit, MI, USA), both supplemented with NaCl to a final concentration of 1% (*w*/*v*). Bacteria in TSB medium were then grown at the same temperature, with continuous agitation (100 rpm) for twenty-four hours. Exponentially growing bacteria were resuspended in sterile PBS and adjusted to 10^8^ colony-forming units (c.f.u.) mL^−1^. The bactericidal activity was determined following the method of Stevens et al. [29] with some modifications using the MTT assay, which is based on the reduction of the soluble yellow tetrazolium salt (3-(4,5-dimethylthiazol-2-yl)-2,5-diphenyltetrazolium bromide) to a blue, insoluble formazan product by the mitochondrial enzyme succinate dehydrogenase [30]. Samples of 20 μL of serum collected from all individuals (n = 6 for each treatment at each time) were added to the wells of a 96-well plate. A PBS solution was added to some wells in place of the samples and served as a positive control. Aliquots of 20 μL of the previously cultured bacteria were added, and the plates were incubated for 5 h at 25 °C. After that, 25 μL of MTT (1 mg mL^−1^) were added to each well, and the plates were again incubated for 10 min at 25 °C to allow formazan formation. The plates were then centrifuged (2000× *g*, 10 min), and the precipitates dissolved in 200 μL of DMSO were transferred to a 96-well flat-bottom plate. The absorbance of dissolved formazan was measured at 570 nm with a plate reader. Bactericidal activity was expressed as the percentage of nonviable bacteria, calculated as the difference between the absorbance of surviving bacteria compared to the absorbance of bacteria from positive controls (100%).

### 2.7. Cellular Immune Parameter

#### 2.7.1. Respiratory Burst Activity

The respiratory burst activity was studied in HK leucocytes by a chemiluminescence method described by Bayne and Levy [31] in all samples (n = 6 for each treatment at each time). Samples of 100 μL of leucocyte suspension were placed in triplicate in the wells of a 96-well flat-bottom plate. Then, 100 μL of HBSS containing 1 mg mL^−1^ phorbol myristate acetate (PMA, Sigma-Aldrich, St. Louis, MO, USA) and 10^4^ M luminol (Sigma-Aldrich, St. Louis, MO, USA) were added to each well. The plate was set in motion and immediately read for 1 h at 2 min intervals. The kinetics of the reactions were analyzed, and the maximum slope of each curve was calculated. Luminescence controls were calculated using reagent solutions containing luminol but not PMA.

#### 2.7.2. Phagocytosis

The phagocytosis by gilthead seabream HK leucocytes was studied in all samples (n = 6 for each treatment at each time) by flow cytometry according to Rodríguez et al. [28]. Briefly, 100 μL of HK leucocytes in sRPMI and 60 μL of *Saccharomyces cerevisiae* (5 × 10^7^ cells mL^−1^ of sRPMI) were mixed and centrifuged (400× *g*, 5 min, 22 °C) before being resuspended and incubated at 22 °C for 30 min. Then, the samples were placed on ice to stop phagocytosis and diluted by 400 μL ice-cold phosphate-buffered saline (PBS). The fluorescence of the extracellular yeast cells was quenched by adding 40 μL ice-cold trypan blue (0.4% in PBS). All samples were analyzed in a flow cytometer to analyze the phagocytic cells (FACsort, Becton Dickinson, San José, CA, USA). The phagocytic ability was defined as the percentage of phagocytic cells with one or more ingested yeast, and phagocytic capacity was the mean fluorescence intensity. Phagocytic ability was defined as the percentage of cells with one or more ingested bacteria (green-FITC fluorescent cells) within the phagocytic cell population, while the phagocytic capacity was the mean fluorescence intensity [32]. 

#### 2.7.3. Peroxidase Activity

The peroxidase activity in leucocytes was measured in all samples (n = 6 for each treatment at each time) according to Quade and Roth [26], as described above. Briefly, 10^6^ HK leucocytes in sRPMI were lysed with 0.002% cetyltrimethylammonium bromide (CTAB; Sigma-Aldrich, St. Louis, MO, USA), and after centrifugation (400× *g*, 10 min, 25 °C), 150 μL of the supernatants were transferred to a fresh 96-well plate containing 25 μL of 10 mM TMB and 5 mM H_2_O_2_. The color change reaction was stopped after 2 min, and the OD was read at 450 nm. Standard samples without leucocytes were used as blanks.

### 2.8. Statistical Analyses 

The results in the figures are expressed as the mean ± standard error (SEM). The normality of the variables was confirmed by the Shapiro–Wilk test and homogeneity of variance by the Levene test. Statistical differences among the groups of treatments were assessed by one-way ANOVA analyses, followed by the Tukey test or Games Howell test, depending on the homogeneity of the variables. The significance level was 95% in all cases (*p* < 0.05). All the data were analyzed by the computer application SPSS for Windows^®^ (version 15.0, SPSS Inc., Chicago, IL, USA).

## 3. Results

### 3.1. Growth Performances 

The growth performance parameters, calculated after 30 days of experimental feeding, are presented in Table 1. Fish of lot D2, fed for 30 days with the highest inclusion of *H. salinarum*, showed a significant increase in body weight, in weight gain (WG) and specific growth rate (SGR) compared to the commercial diet (Control) and D1 diet (*p* < 0.05).

### 3.2. Humoral Immune Parameters

**Figure 1:**
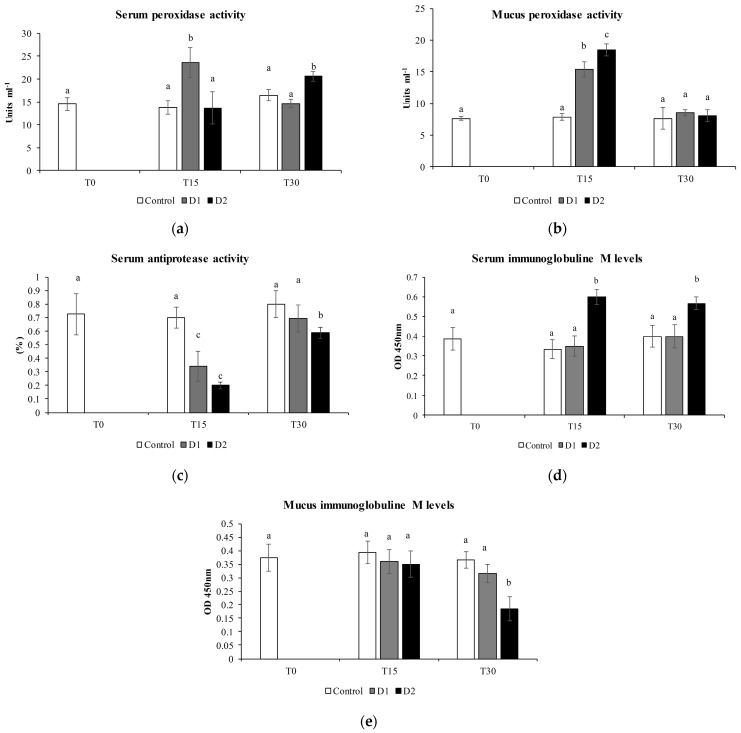
(**a**) Serum peroxidase activity (units mL^−1^). (**b**) Mucus peroxidase activity (units mL^−1^), (**c**) serum antiprotease activity (%), (**d**) serum immunoglobulin M levels (OD 450 nm) and (**e**) mucus immunoglobulin M levels of gilthead seabream specimens fed different diets for 15 days (T15) and 30 days (T30). Control diet (white bars), 0.05% inclusion of *H. salinarum* (D1, grey bars) and 0.1% inclusion of *H. salinarum* (D2, black bars). Values are expressed as the means ± SEM (n = 6). Statistical differences (*p* < 0.05) among groups are indicated by different superscripts letters.

Among humoral immune parameters, the determination of the peroxidase activity in the serum showed a significant increase at 30 days in fish fed D2 diet, with the highest inclusion of *H. salinarum*, with respect to the control and D1 treatment (*p* < 0.05) (Figure 1a). In the mucus, this activity presented a significant increase at 15 days in fish fed the D1 diet (Figure 1b) but not at 30 days. The protease activity did not show statistically significant differences among treatments, while the antiprotease activity in the serum showed a significant reduction at 15 days in both diets with respect to the control (*p* < 0.05) and increased after 30 days of the experimental diet (Figure 1c). The IgM levels showed a significant increase after 30 days in the serum of fish fed experimental diet D2, with the highest inclusion of *H. salinarum*, compared to the control (*p* < 0.05) (Figure 1d), while, in the skin mucus, the IgM levels were reduced only at the highest inclusion of the (D2) after 30 days of administration of the experimental diet (Figure 1e).

### 3.3. Bactericidal Activity 

**Figure 2:**
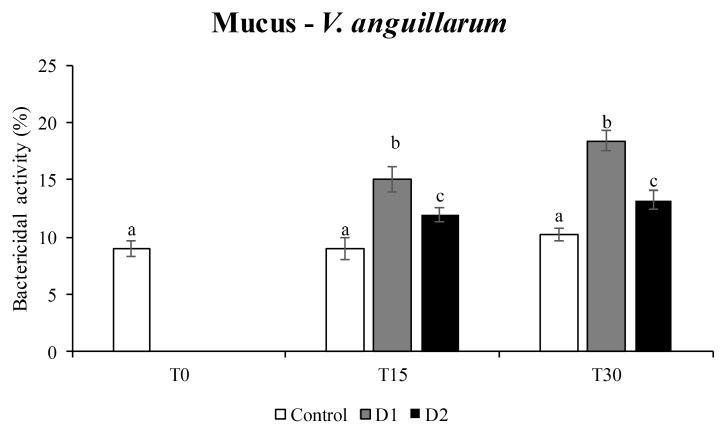
Bactericidal activity against *Vibrio anguillarum* determined in the mucus of gilthead seabream specimens fed different diets for 15 days (T15) and 30 days (T30). Control diet (white bars), 0.05% inclusion of *H. salinarum* (D1, grey bars) and 0.1% inclusion of *H. salinarum* (D2, black bars). Values are expressed as the means ± SEM (n = 6). Statistical differences (*p* < 0.05) among groups are indicated by different superscripts letters.

The results of the bactericidal activity showed a significant efficacy of the supplemented diets with *H. salinarum* at both doses (D1 and D2) only in the mucus against *V. anguillarum* (Figure 2), while no differences were recorded against *V. harveyi*.

### 3.4. Cellular Immune Parameter

Among the cellular immune parameters, the respiratory burst activity showed a significant increase compared to the commercial diet (control) (*p* < 0.05) after 15 days of administration of both experimental diets supplemented with the two levels of *H. salinarum* (Figure 2a). After 30 days, the respiratory burst activity showed a reduction similar to the control for D1 and a significant decrease for D2. The phagocytic ability and myeloperoxidase activity showed an increase with respect to the control after 30 days of administration of the two experimental levels of inclusion (*p* < 0.05) (Figure 2b,c).

## 4. Discussion

In aquaculture sector, the interest in the use natural compounds to promote health, welfare and immunity in farmed fish is growing [8,33,34,35,36]. Among natural compounds, the use of probiotics is significantly increasing, and a growing number of studies are demonstrating their positive effects in the most economically important fish species [37,38,39]. Potential probiotic bacteria and other microorganisms, such as yeasts and fungi, are able to synthesize a wide spectrum of bioactive compounds, from hexopolysaccarides to antioxidants, responsible for their beneficial properties on farmed fish immunostimulation [40].

It is known that different *Bacillus* species have an adhesion capacity, produce bacteriocins (antimicrobial peptides) and have an immunostimulatory effect [41,42].

In cod and herring larvae, bacterial ingestion and subsequent endocytosis have been shown to be involved in the stimulation of the developing immune system [43]. 

In our study, the obtained results showed the best growth performances in seabream fed with a high inclusion of *H. salinarum* in the diet (0.1%) both at 15 and 30 days (Table 1), suggesting that higher doses and the time of administration of this potential probiotic species could provide greater benefits in terms of growth. This result could be due to the ability of probiotics to colonize the intestinal tract, facilitating the assimilation of nutrients and, especially, having an anti-inflammatory effect on the intestine. Numerous studies have shown that the administration of probiotics often results in improved growth performance; specifically, probiotics may act directly by increasing the appetite and growth regulation or indirectly through improved digestibility [44]. 

In general, positive growth performances followed the administration of natural compounds and are considered a positive effect of the supplementation on fish growth [45,46], suggesting that fish can utilize dietary nutrients more efficiently when feed is enriched with natural compounds [13]. Several authors have pointed out that one of the main modes of action of the beneficial effects of probiotics in reared organisms is the production of additional digestive enzymes, which results in increased growth and feed efficiency and the prevention of intestinal disorders of the host species [13,34,47]. In fish, innate and adaptive immunity are commonly divided into three components: humoral parameters cellular components and the epithelial/mucosal barrier [48]. The innate immune response in fish is essential to fight pathogens due to the limitations of the adaptive immune system. However, each component of the immune system has its own value, and the final combination will result in a satisfactory immune response [49]. 

Several studies have shown that the dietary administration of probiotics stimulates both the innate and adaptive immune mechanisms in fish [23,24]. Protease and antiprotease activities are related to the defenses against bacterial or parasitic infections [50]. The skin mucus, a key component of fish immunity, contains a large number of innate immune components, such as lysozymes, proteases and proteolytic enzymes [51]. Proteases are secreted in the skin mucus as a response to pathogen recognition. They effectively prevent pathogen invasion and facilitate the removal of pathogens from body surfaces by activating the innate and adaptive immune systems [45]. In fact, proteases can act directly against the pathogens through proteolysis to kill bacteria or modify the mucus consistency or increase mucus shedding. In this study, protease activity did not differ significantly between the control and experimental diets with *H. salinarum* inclusion; on the contrary, the antiprotease activity, after a significant decrease after 15 days of administration of the supplemented diet (Figure 1), returned to the levels comparable to the control at 30 days. Antiprotease activity is normally high in fish and barely altered even after immunization or infection [35], although it is reported that certain probiotics can modulate this activity in fish [52,53]. To date, very few data are available, but this might be interesting, since they inhibit the action of proteases of some pathogenic bacteria [53], although more research is needed to elucidate this issue.

Probiotics envisage multiple modes of action in fish and, among these, are able to exert increase the production of antibodies, macrophages, lysozyme, etc. [10]. Antibodies (immunoglobulins) are the principal components of the humoral adaptive immune system in all classes of vertebrates, and IgM are the main effector molecules of the teleost immune system, found in blood and other body fluids, such as mucus, and is the main player in systemic immune responses [54]. The overall results of the present study did not reveal significant variations in the mucus while showing a significant increment at the highest dose of the serum starting from 15 days of administration (Figure 1d), which could be explained with the role of probiotics in stimulate immunoglobulin production [10]. Along with the probiotic role, stimulation could be also explained by the presence of carotenoids in *H. salinarum*, as in other studies that have employed antioxidants as additives which stimulated the secretion of IgM [55].

Bactericidal activity is an important immune response involved in the suppression of pathogenic microbes in fish [33]. According to Chuphal et al. [10], the probiotic role of Archaea is also attested by the ability to reduce or diminish the virulence of pathogens thanks to various antimicrobial (antibacterial, antiviral, antifungal, etc.) properties. In our study, bactericidal activity was tested against two different pathogens dangerous for farmed fish: *V. harveyi* and *V. anguillarum*. The obtained results attested a significant efficacy of the supplemented diets with *H. salinarum*, at the lower dose, against *V. anguillarum* (Figure 2). In fish, it has been shown that some natural substances contained in probiotics, mainly polysaccharides and antioxidants, can increase the protection against certain diseases, counteracting microorganisms. To overcome the use of antibiotics to control diseases in aquaculture, the use of probiotics and bioactive molecules as immunostimulants is gaining great attention due to their less toxic, ecofriendly nature and their ability to activate the pattern recognition receptors of the host immune system, resulting in an immune response that increase the resistance. 

To complete the study of the fish immune status, the cellular immunity was measured in gilthead seabream leucocytes taken from an encephalic kidney. Leukocyte respiratory burst activity is achieved by the production of reactive oxygen species by neutrophils, monocytes, macrophages, dendritic cells and B lymphocytes as a strategy of the immune system for several pathogens’ destruction.

The ‘oxidative burst’ is a resting metabolic pathway in cells; however, it is activated after microbial agent detection. It has the function of producing strong oxidizing compounds (burst activity) that act to destroy microorganisms [56,57,58,59].

Our results are consistent with others that have reported that probiotics and their bioactive compounds, such as carotenoids and other antioxidants, are able to stimulate fish immune systems [32].

**Figure 3:**
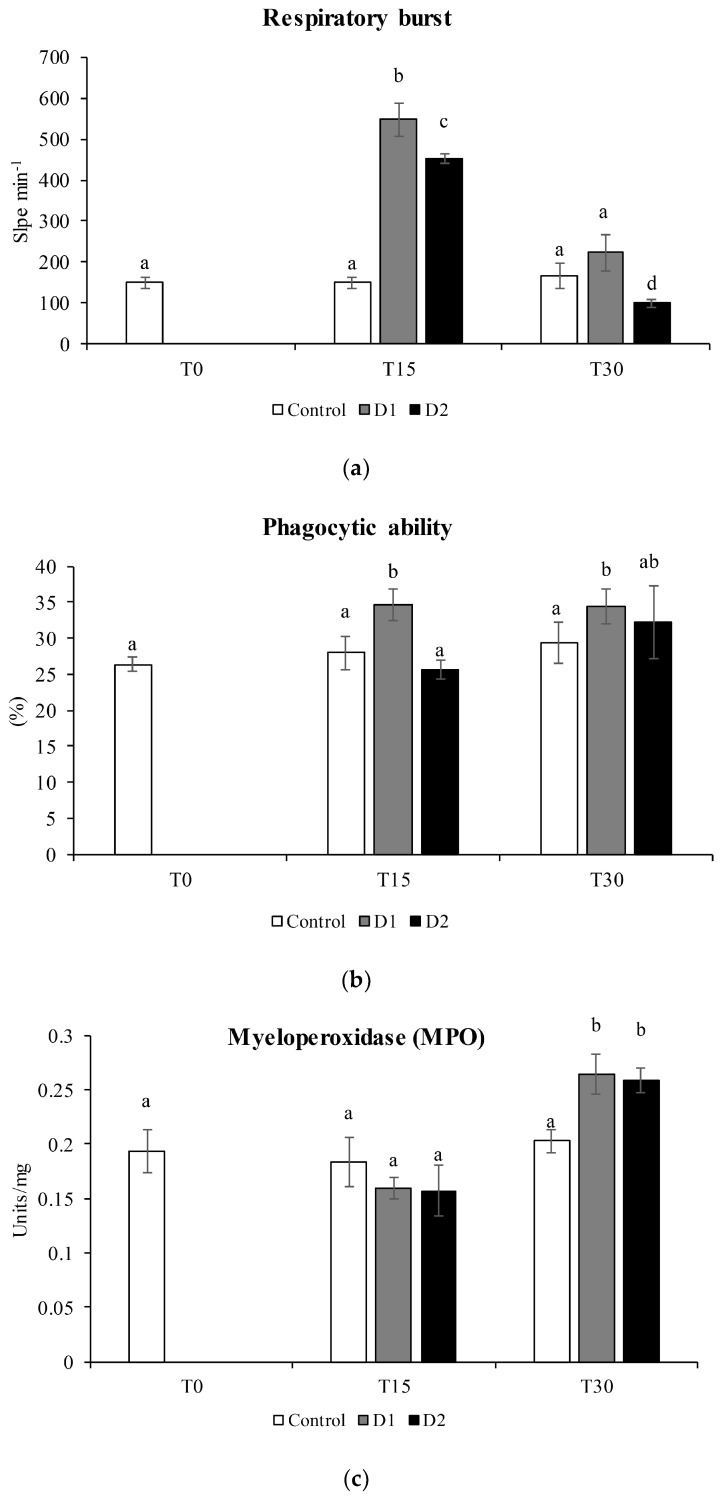
(**a**) Respiratory burst (slope min^−1^). (**b**) Phagocytic ability (%). (**c**) Myeloperoxidase in head kidney leucocytes of gilthead seabream specimens fed different diets for 15 days (T15) and 30 days (T30). Control diet (white bars), 0.05% inclusion of *H. salinarum* (D1, grey bars) and 0.1% inclusion of *H. salinarum* (D2, black bars). Values are expressed as the mean ± SEM (n = 6). Statistical differences (*p* < 0.05) among groups are indicated by different superscripts letters.

We observed a positive effect in the first 15 days of treatment and a normalization after 30 days (Figure 3); this could be explained by normalization of the respiratory burst parameters. These results are in agreement with the literature, as reported by several authors, who suggested that an increase in respiratory burst activity is a general characteristic of probiotics [60,61,62]. 

Phagocytosis, considered the most prominent leucocyte activity of innate immunity [46], increased in fish fed the experimental diets after 15 days, but the increase was not maintained at 30 days. These results agree with the immunostimulants effects determined by the use of natural compounds as supplements for fish diets [55]. Our data suggest that *H. salinarum* has an immunostimulant effect after 15 days of administration, but these effects disappear after 30 days as an adaptive response (Figure 3). 

From the various studies, it has been noted that the phagocytic capacity is one of the most important cellular responses of leukocytes in innate immunity. Myeloperoxidase is an important enzyme that has the ability to kill pathogens [63]. This enzyme showed higher values after 30 days of treatment (Figure 3), suggesting that this strain has immunostimulatory properties for the cellular activities of the head kidney and, therefore, can stimulate an immune response.

## 5. Conclusions

The overall results of the present study showed that the inclusion of *H. salinarum* in the diet of gilthead seabream could exert some beneficial effects, as attested by the growth promotion, humoral and cellular immunity and bactericidal activity. In particular, the highest level of inclusion at D2 induced an amelioration in the growth performance with respect to the Control and D1 and a significant increment of serum IgM, which could be explained with the role of probiotics in stimulating immunoglobulin production and by the presence of carotenoids in *H. salinarum*.

As it regards the cellular immunity response, the results highlighted an increase in respiratory burst activity, which is a general characteristic of probiotics and immunostimulants. 

The phagocytic capacity, one of the most important cellular responses of leukocytes’ innate immunity, and myeloperoxidase, an important enzyme to kill pathogens, showed higher values with respect to the control, suggesting that this strain has immunostimulatory properties for the cellular activities of the head kidney and, therefore, can stimulate the immune response. Finally, the obtained results attested a significant efficacy of the supplemented diets with *H. salinarum*, at the lower dose, against *V. anguillarum*.

However, these beneficial effects need to be supported by other investigations and analyses, such as the microbiota, which could expand the amount of data necessary to attest a realistic beneficial effect in the diet. If these results are confirmed by our next experiment, Archaea can be an alternative to existing feed probiotics due to their heat stability without compromising any basic probiotic features [10].

## Figures and Tables

**Table 1:** Growth performances determined in gilthead seabream after 30 days of experimental feeding with a commercial diet (Control) and diets supplemented with 0.05% (D1) and 0.1% (D2) inclusion of *H. salinarum*. Values are the mean ± SEM (n = 6). Statistical differences (*p* < 0.05) among groups are indicated by different superscripts letters.

	Control	D1	D2
Weight	11.48 ± 1.9 ^a^	13.18 ± 0.83 ^a^	15.11 ± 1.1 ^b^
WG	68.33 ± 1.70 ^a^	72 ± 1.24 ^a^	77.44 ± 1.43 ^b^
SGR	1.89 ± 0.78 ^a^	3.95 ± 0.86 ^ab^	5.80 ± 0.87 ^b^

## Data Availability

The data may be provided under reasonable request.
